# Halide exchanged Hoveyda-type complexes in olefin metathesis

**DOI:** 10.3762/bjoc.6.125

**Published:** 2010-11-23

**Authors:** Julia Wappel, César A Urbina-Blanco, Mudassar Abbas, Jörg H Albering, Robert Saf, Steven P Nolan, Christian Slugovc

**Affiliations:** 1 Institute for Chemistry and Technology of Materials (ICTM), Graz University of Technology, Stremayrgasse 9, 8010 Graz, Austria; 2 EaStCHEM School of Chemistry, University of St Andrews, St Andrews KY16 9ST, United Kingdom

**Keywords:** cross metathesis, olefin metathesis, RCM, ROMP, ruthenium

## Abstract

The aims of this contribution are to present a straightforward synthesis of 2^nd^ generation Hoveyda-type olefin metathesis catalysts bearing bromo and iodo ligands, and to disclose the subtle influence of the different anionic co-ligands on the catalytic performance of the complexes in ring opening metathesis polymerisation, ring closing metathesis, enyne cycloisomerisation and cross metathesis reactions.

## Introduction

**Figure 1:**
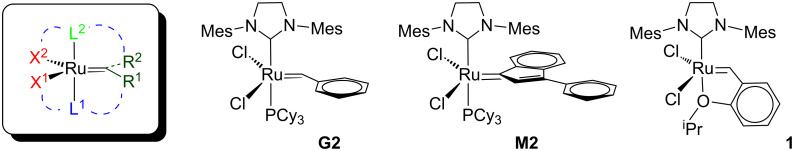
General layout for modifications of ruthenium-based olefin metathesis catalysts (red: anionic ligands; green: nondissociating ligand, e.g. NHC; blue: leaving group, e.g. phosphine or pyridine; olive: carbine substituents; and dashed lines symbolise possibilities of chelation). Three commercial and frequently used catalysts (**G2**: Grubbs 2^nd^ generation catalyst; **M2**: Neolyst M2; and **1**: Hoveyda 2^nd^ generation catalyst).

Since the pioneering reports on the utilisation of *N*-heterocyclic carbenes (NHC) as co-ligands in ruthenium-based carbene complexes for olefin metathesis [1–3] in the late nineties of the last century, olefin metathesis has become a powerful carbon-carbon double-bond-forming tool presenting unique synthetic opportunities [4]. Developments in this area can be attributed to a steady and competitive research, focused on improving activity, selectivity and functional group tolerance of the catalysts by changing the leaving co-ligand [4–5], by using tailored carbene ligands [5–7], by introducing new NHC ligands [5,8–9], or by variation of the anionic co-ligands [5] (Figure 1).

Compared with other modifications, little attention has been paid to the exchange of anionic co-ligands. In most cases chloro ligands have been exchanged for sulfonates or fluorocarboxylates [10], often with the aim to heterogenise the catalysts [11], but also phenolates [12–13] and pseudohalides [14] as well as halides other than chloride [15–19]. An early study dealing with the change of reactivity upon exchanging the chloride ligands in **G2** for bromides and iodides showed increasing initiation rates (phosphine dissociation is facilitated), but decreasing propagation rates with increasing steric bulk of the halides [15]. Iodide bearing catalysts have been successfully used in asymmetric olefin metathesis reactions, where they show, in most cases, better enantio- or diastereo-selectivity compared to their chloride counterparts, but at the price of lower activity [16–19]. As shown by Braddock et al., halides and more generally various anionic ligands are labile in solution, and these complexes undergo anionic ligand exchange even in nonprotic media at room temperature [20]. This particular result is an important consideration whenever charged substrates are transformed.

The lack of reactivity data for halide-exchanged complexes prompted us to investigate the catalytic activity of bromo and iodo analogues of Hoveyda 2^nd^ generation catalyst (**1**) in ring closing metathesis (RCM), enyne metathesis and cross metathesis (CM). Moreover, the scope of these compounds in ring opening metathesis polymerisation (ROMP) [21] was also studied.

## Results and Discussion

### Synthesis and characterisation

**Scheme 1:**
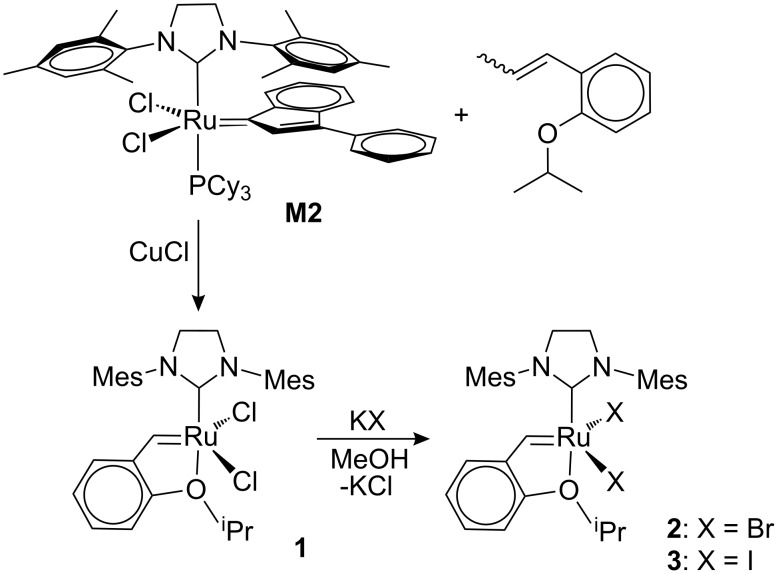
Synthesis of **1**, **2** and **3**.

Although complex **1** is commercially available, we prepared **1** from (H_2_IMes)(PCy_3_)Cl_2_Ru(3-phenyl-indenylid-1-ene) (**M2**) as the ruthenium-containing starting material (Scheme 1). **M2** is a relatively more economic alternative to **G2**, bearing an indenylidene instead of a benzylidene ligand [22–24]. Adopting Hoveyda’s protocol for obtaining **1** from **G2** [25] and using 1-isopropoxy-2-(prop-1-en-1-yl)benzene as the carbene precursor, **1** can be obtained in 78% yield. Complexes **2** and **3** were prepared by addition of excess potassium bromide (KBr) or potassium iodide (KI) to a suspension of **1** in methanol, following the procedures for similar transformations of different dichloro carbene complexes to their diiodo analogues [26]. In these cases THF [15,26] or acetone [27] rather than methanol were used as the solvents.

**Figure 2:**
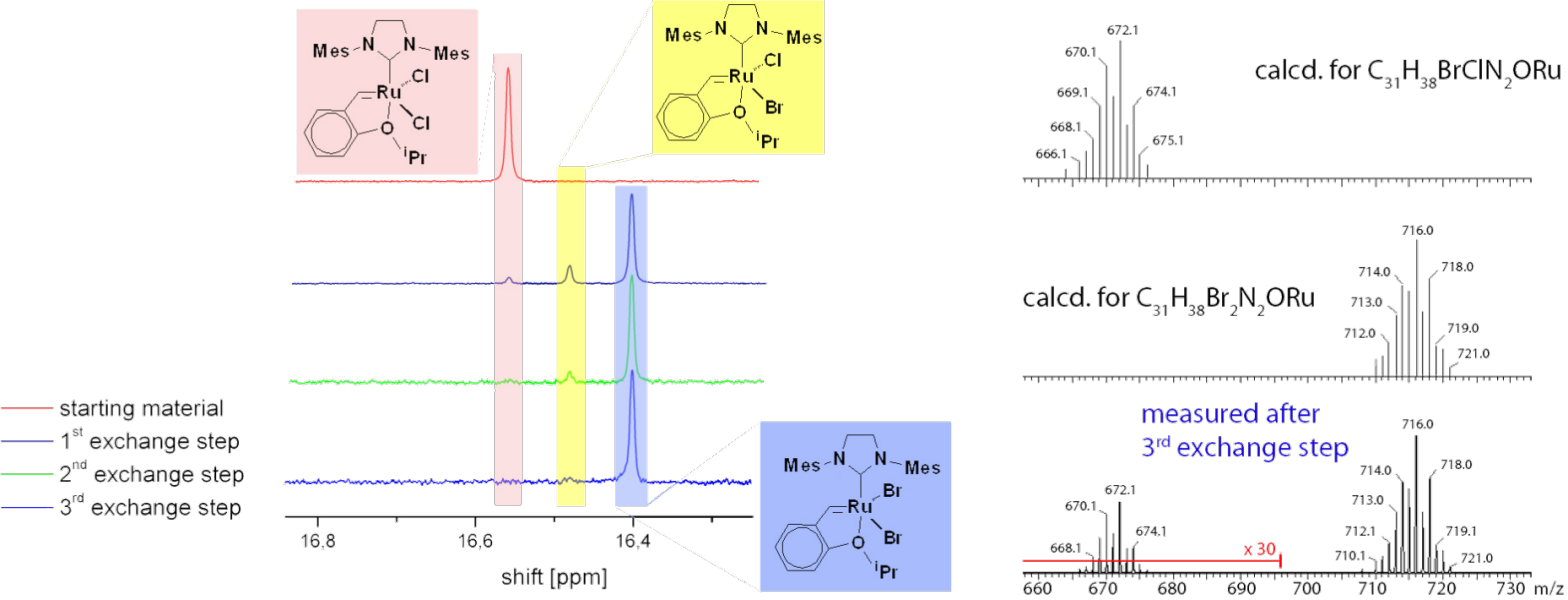
Details of the ^1^H NMR spectra acquired during the synthesis of **2** and the FD-MS spectrum of **2** isolated after the 3^rd^ exchange step.

The halogen exchange reaction proved rapid at room temperature and reached an equilibrium comprising of three different species within less than 1 h. The compounds were identified as the starting material **1**, the desired product **2** (or **3**), and a “mixed halogen” compound bearing a chloride and a bromide or an iodide ligand, respectively (Figure 2). Upon removal of the inorganic salts and addition of a further portion of KBr or KI, the equilibrium can be directed towards the desired product. Typically, three successive additions of the potassium salt are necessary to obtain **2** or **3** in 90–92% yield and 95–98 % purity. Efforts to further shift the equilibrium towards **2** or **3** have so far proved unsuccessful. The impurity, which could not be separated by recrystallisation or column chromatography, was identified as the “mixed halogen” compound and as revealed by field desorption mass spectrometry (FD-MS) measurements. FD-MS was found to be a suitable technique for the characterisation of this type of complex. Selecting appropriate acquisition parameters – the emitter current was slowly increased until desorption/ionisation started, in this way only molecular ions M^+^ were observed (Figure 2).

Quantification was carried out by integration of the corresponding ^1^H NMR signals. ^1^H NMR spectra allowed convenient monitoring of the halide exchange by observing the carbene region at the very low field region of the spectra. The starting complex **1** exhibits a carbene peak at 16.56 ppm. Exchange of both chloride ligands for bromide shifts the carbene peak upfield to 16.40 ppm and the mixed chloro-bromo complex appears at 16.48 ppm. In the case of **3**, the carbene proton exhibits a singlet at 15.66 ppm and the chloro-iodo species displays the corresponding peak at 16.10 ppm. All other features of the ^1^H NMR spectrum of **2** are similar to those of **1** indicating slightly hindered rotation of the *N*-heterocyclic carbene ligand and a *trans*-disposition of the two halide ligands. In contrast, the rotation of the NHC ligand around the Ru–NHC bond in **3** is hindered as shown by a magnetic non-equivalency of the signals corresponding to the two mesityl moieties. The same behaviour was observed in the corresponding ^13^C NMR spectra (Supporting Information File 1).

### X-Ray

**Figure 3:**
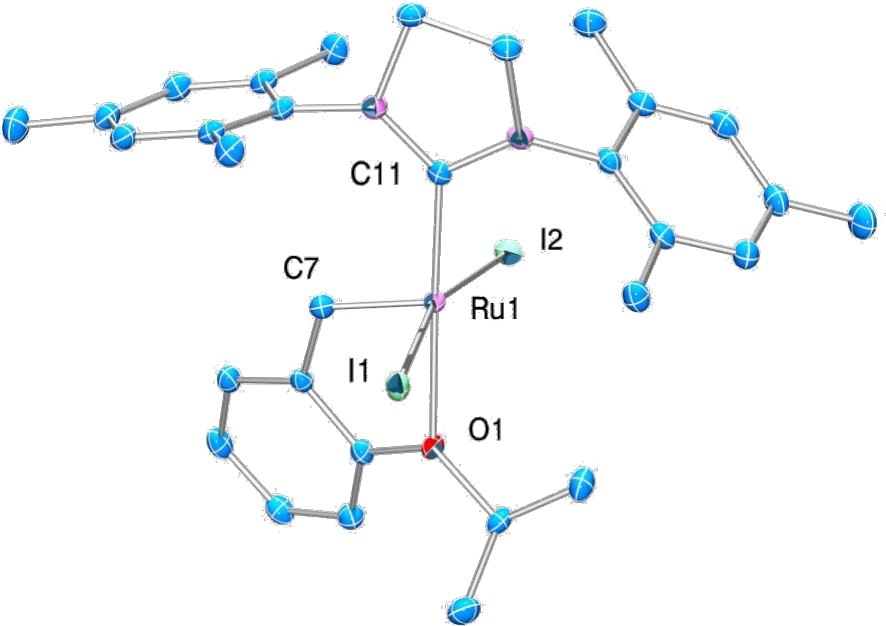
ORTEP drawing of **3**. Thermal ellipsoids are drawn at 50% probability level. Hydrogen atoms are omitted for clarity.

Compound **3** crystallises in the monoclinic space group *P*2_1_/*c*, and the overall geometrical arrangement is isostructural to the parent Hoveyda complex **1** (Figure 3). The ruthenium atom is pentacoordinated; the ligands form a slightly distorted square pyramid. The two iodides are, as expected, as supported by NMR data, *trans*-oriented in the basal plane of the square pyramid. The other positions in the basal plane are occupied by C11 (of the NHC ligand) and the atom O1. The strong ruthenium–carbon bond to the carbene was found in the apical position of the square pyramidal coordination around the metal center. Selected bond lengths and angles are provided in Table 1. The overall geometry around the transition metal centre and most of the bond lengths in **3** are analogous to their related values in complex **1**. This is surprising since the Ru–I bond lengths are considerably longer compared to the Ru–Cl bonds in **1**. The bond angles vary slightly due to the significantly larger ionic radius of the iodide ligands [28], which lead to a slight distortion of the complex compared to the chloride-bearing compound.

**Table 1:** Comparison of bond lengths and angles in **1** and **3**.

**Bond**	**Bond length in 3 [Å]**	**Bond length in 1 [Å]^a^**

Ru–C11	1.982	1.981
Ru–C7	1.834	1.834
Ru–O	2.282	2.261
Ru–X1	2.677	2.340
Ru–X2	2.663	2.328

**Angle**	**Bond angle in 3 [°]**	**Bond angle in 1 [°]^a^**

C7–Ru–C11	102.94 (7)	101.5 (14)
C7–Ru–O	78.82 (6)	79.3 (17)
C11–Ru–O	178.13 (5)	176.2 (14)
C7–Ru–X2	96.07 (5)	100.2 (15)
C11–Ru–X2	96.08 (4)	96.6 (12)
O–Ru–X2	84.35 (3)	86.9 (9)
C7–Ru–X1	96.70 (5)	100.1 (15)
C11–Ru–X1	90.78 (4)	90.9 (12)
O–Ru–X1	88.35 (3)	85.3 (9)
X2–Ru–X1	163.78 (6)	156.5 (5)

^a^Taken from Ref. [25]

Although the overall structure is quite similar to **1**, some parameters concerning the ruthenium environment are worth discussing in more detail. As expected the main difference appears in the ruthenium halide bond lengths (in case of **3** about 0.3 Å longer) and in the I–Ru–I angle (enlarged by some 7°). Both, the longer bond distance and the enlarged angle, are caused by the larger ionic radii of the iodides. The fact that the Ru–C and Ru–O distances are not significantly affected by the larger ionic radius of the halide ligands can be easily understood by considering the structural flexibility of the coordination polyhedron around the ruthenium atom. The X1–Ru–X2 angle has a relatively high degree of freedom as the opposed position to the apical Ru–C bond is not occupied, and thus the halide ions can avoid close contact with other ligands – which would distort the complex severely – by shifting their positions towards (chloride) or away from (iodide) the empty coordination position, depending on the Ru–X distances.

### Catalytic testing of the compounds

#### ROMP

**Figure 4:**
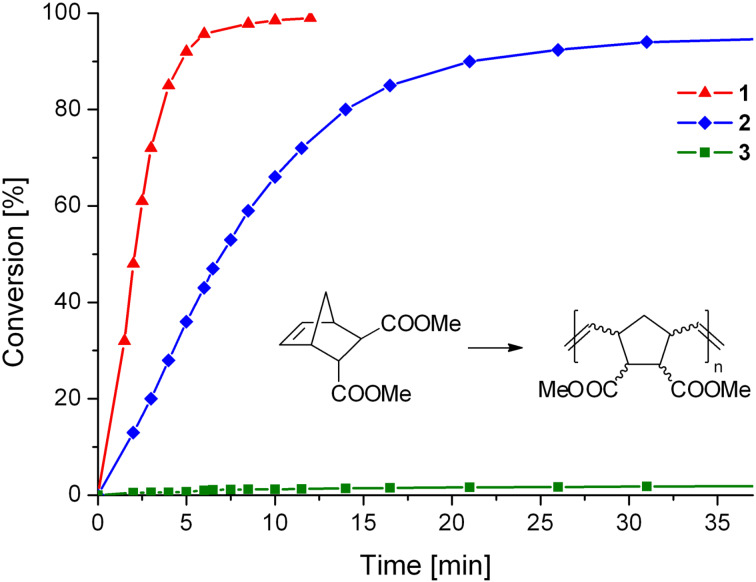
Polymerisation of **4** as a function of time, initiated by **1**, **2** or **3**, monitored by ^1^H NMR spectroscopy; Reaction conditions: **4**:initiator = 50:1; [**4**] = 0.1 mol/L; solvent: CDCl_3_; 20 °C.

Initiators **1**–**3** were benchmarked in the ROMP of dimethyl bicyclo[2.2.1]hept-5-ene-2,3-dicarboxylate (**4**). The conversion of the monomer was monitored using arrayed ^1^H NMR spectroscopy (Figure 4). Initiator **1** yields complete conversion of **4** at 20 °C in about 10 min (half-life t_½_ ≈ 2 min), while the dibromo derivative **2** requires about 35 min (t_½_ ≈ 7 min) for complete consumption of the monomer. Complex **3** is almost unable to initiate ROMP of **4** at room temperature.

Additional polymerisation tests were carried out using standard conditions [29], and, in addition to **4**, two further monomers, namely 5,6-bis(methoxymethyl)bicyclo[2.2.1]hept-2-ene (**5**) and (*Z*)-cyclooct-5-ene-1,2-diyl diacetate (**6**), were used. Polymers made of **4** and **5** are not prone to backbiting, i.e., no secondary metathesis reaction affects the double bonds of the formed polymer. Therefore the average number molecular weight (*M*_n_) can be used to establish an indirect, qualitative comparison of the ratio of initiation rate to propagation rate (*k*_i_/*k*_p_) of a given initiator and monomer combination [30]. Polymers made with **M2** and **M31** were used for further comparison. **M2** (*k*_i_/*k*_p_ ≈ 1–0.01) is a typical initiator, producing in most cases polymers with high *M*_n_ values and high polydispersity indices (PDI) (Table 2, Entry 1 and 7), while polymers prepared with **M31** (*k*_i_/*k*_p_ ≈ 10–1000) are typically characterised by low *M*_n_ values and low PDIs [24] (Table 2, Entry 2 and 8).

Polymerisations initiated with the dichloro derivative **1** yield polymers with relatively low *M*_n_ and fairly narrow molecular weight distributions (Table 2, Entry 3 and 9), meaning that *k*_i_ is higher than *k*_p_ although both values are of the same order of magnitude. In the case of monomer **4**, *k*_i_/*k*_p_ increases upon changing from the chloro to the bromo ligands as can be deduced from the lower *M*_n_ value of the resulting polymer (68500 g/mol in case of **2** and 106000 g/mol in case of **1** as the initiator). As can be seen in Figure 4, the polymerisation with initiator **2** is distinctly slower than for the one initiated with **1**, meaning that *k*_p_ for a polymerisation system consisting of **1** and **4** is distinctly higher than *k*_p_ for **2** and **4**. Diiodo-bearing initiator **3** failed in the polymerisation of **4** at room temperature, but gave 75% conversion upon heating in toluene at 80 °C for 19 h, meaning that *k*_p_ is very low in this system. In summary, the following qualitative trends for the polymerisation of **4** with initiators **1**, **2** and **3** could be established: the propagation rate constant decreases with increasing steric demand of the halo ligands (i.e., *k*_p_(**1**) > *k*_p_(**2**) >> *k*_p_(**3**)) and the ratio of initiation rate to propagation rate increases on going from **1** to **2** (i.e., *k*_i_/*k*_p_(**1**) < *k*_i_/*k*_p_(**2**) ≈ *k*_i_/*k*_p_(**3**) > 1) but remains of the same order of magnitude.

**Table 2:** Polymerisation results^a^.

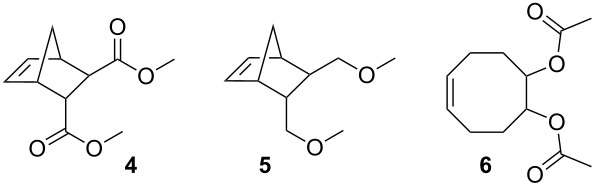							

Entry	Monomer	Initiator	Time [min]	Conversion [%]	Yield [%]	*M*_n_^b^ [kg/mol]	PDI^b^

1^c^	**4**	**M2**	300	100	89	654	2.7
2^c^	**4**	**M31**	80	100	72	45.5	1.08
3	**4**	**1**	80	100	85	106	1.2
4	**4**	**2**	80	100	79	68.5	1.3
5	**4**	**3**	1080	3	—	—	—
6^d^	**4**	**3**	1140	75	47	53.1	2.3
7^c^	**5**	**M2**	360	100	87	967	2.3
8^c^	**5**	**M31**	90	100	74	64.7	1.09
9	**5**	**1**	80	100	87	65.7	1.2
10	**5**	**2**	80	100	77	75.3	1.5
11	**5**	**3**	1080	78	44	82.8	8.8
12^d^	**5**	**3**	135	90	67	73.3	2.3
13	**6**	**1**	75	95	54	130^e^	5.2
14	**6**	**2**	240	92	60	220^e^	1.9
15	**6**	**3**	2880	58	37	190^e^	2.8

^a^Reaction conditions: Monomer:Initiator = 300:1; [monomer] = 0.2 mol/L; solvent: CH_2_Cl_2_; 20 °C.; ^b^determined by GPC, solvent THF, relative to polystyrene standards; ^c^values taken from Ref. [30]; ^d^solvent: toluene, temperature: 80 °C; ^e^additionally a second peak with a *M*_n_ of about 1000 g/mol was observed.

By studying the polymerisation of monomer **5** with **1**, **2** and **3**, a slightly different picture emerged. While the trend for *k*_p_ is the same as in the case of monomer **4**, *k*_i_/*k*_p_ decreases with increasing steric bulk of the halo ligands i.e., *k*_i_/*k*_p_(**1**) > *k*_i_/*k*_p_(**2**) > *k*_i_/*k*_p_(**3**), meaning that the decrease of k_i_ within the series is more pronounced than the decrease of *k*_p_.

At this stage a comment on the presence of the small amounts of mixed halogen complexes (Br–Cl–Ru and Cl–I–Ru both < 5%) is necessary. These species might be responsible for the somewhat higher PDIs of the polymers prepared with **2** compared to those prepared with **1**. Still it can be ruled out that the mixed halogen species is the only active initiator (otherwise the low *M*_n_ values observed for the polymers would not be explicable). Accordingly, the activity of the corresponding mixed halogen species is similar to the activity of **2** or **3**, respectively.

In contrast to monomers **4** and **5**, monomer **6** gives polymers which can be degraded by secondary metathesis reactions [31]. Complex **1** polymerises 300 equiv of **6** in 75 min at room temperature with a conversion of 95% (54% isolated yield). The *M*_n_ of this polymer was 130600 g/mol. Initiator **2** requires 4 h to achieve a conversion higher than 90% (60% yield) and the corresponding *M*_n_ is 220000 g/mol. Finally, **3** gave only 58% conversion after a reaction time of 48 h (*M*_n_ = 190000 g/mol). From these data, it is evident that *k*_p_ decreases within the series **1**, **2** and **3**, and that *k*_i_/*k*_p_ in the case of **6** is considerably smaller when compared to the monomers discussed above.

**Figure 5:**
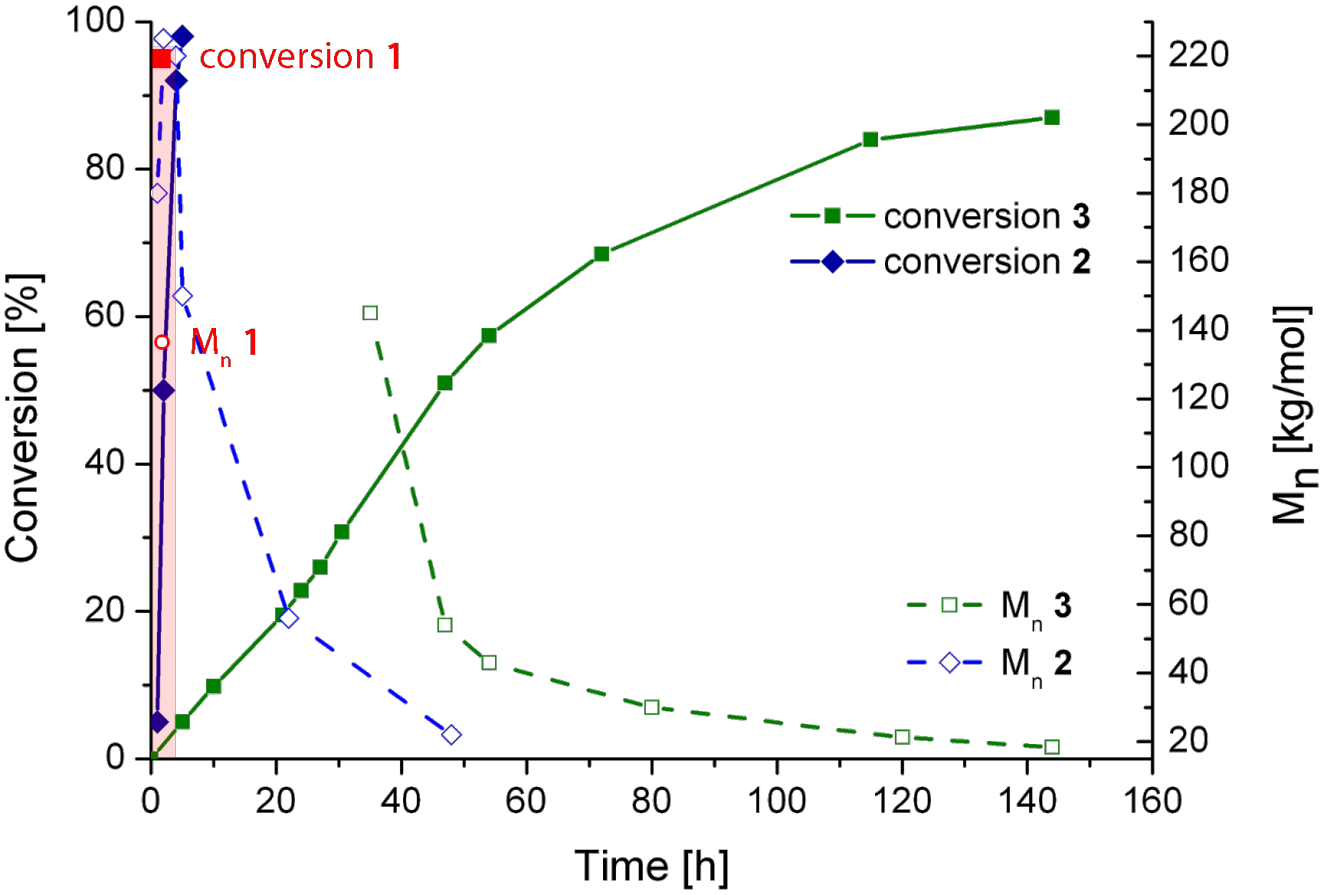
Polymerisations of **6** as a function of time, initiated by **1**–**3**, monitored by ^1^H NMR spectroscopy (solid lines) and GPC (dashed lines); reaction conditions: **6**:initiator = 300:1; [**6**] = 0.2 mol/L; solvent: CH_2_Cl_2_; 20 °C. The red circle symbolises the number molecular weight of **poly6** initiated by **1** (the red square symbolises the conversion after 1 h reaction time); reaction conditions for the polymerisation with **3** is 6:3 = 100:1; [**1**] = 0.05 mol/L; solvent: CH_2_Cl_2_; 20 °C).

In all cases, **poly6** degraded over time (Figure 5), i.e., the overall *M*_n_ decreases after a certain point and broad multimodal molecular weight distributions are observed. In the case of **1** and **2**, the rate of degradation is relatively low when compared to the rate of polymerisation, allowing for the preparation of high molecular weight **poly6** combined with high conversion in short reaction times. In contrast, in the case of **3**, degradation is an important issue and **poly6** of high molecular weight, formed at the early stages of the polymerisation, is substantially degraded long before the remaining monomer is completely consumed.

#### RCM, enyne cycloisomerisation and cross metathesis

**Figure 6:**
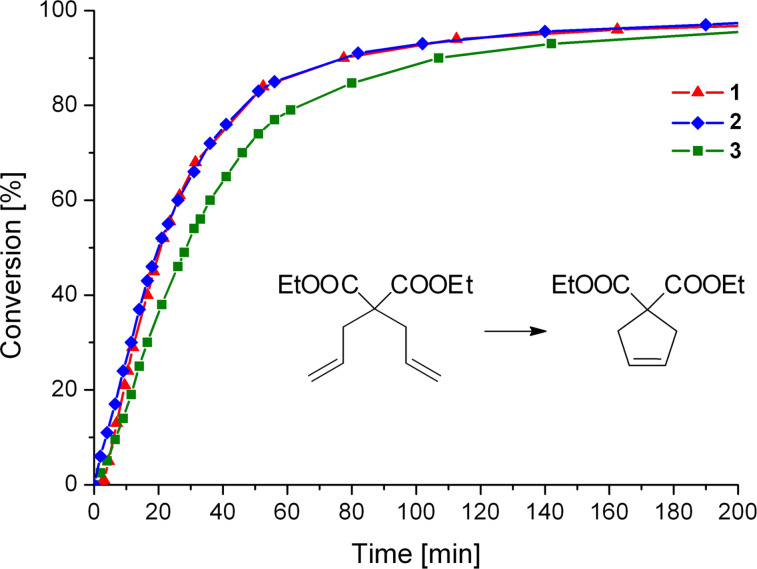
The RCM reaction of **7** as a function of time, catalysed by **1**, **2** or **3**, monitored by ^1^H NMR spectroscopy; Reaction conditions: **7**:catalyst = 100:1; [**7**] = 0.1 mol/L; solvent: CDCl_3_; 20 °C.

Catalytic activities of **1**, **2** and **3** were then evaluated in RCM of diethyl diallylmalonate (**7**). The reaction progress is shown in Figure 6 (for details see Table 3). While **1** and **2** perform equally, **3** is the slowest catalyst for this transformation. Nevertheless, the performance of **3** is, when taking the results from the benchmarking in ROMP into account, remarkable. Complex **3** is a fairly good catalyst for this easy transformation and outperforms **M2** [32].

With these results at our disposal, we concentrated on further elucidating the catalytic potential of **3** in RCM, enyne cycloisomerisation and cross metathesis (CM).

**Table 3:** Results of the catalytic testing.

Entry	Substrate	Product	cat	Reaction conditions	Conv. [%]

1	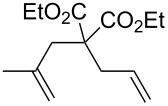	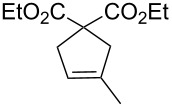	**3** **1**	1 mol %; CH_2_Cl_2_; 20 °C; 24 h	93 >99
2	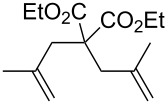	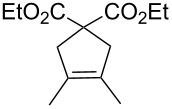	**3** **1**	5 mol %; toluene; 80 °C; 5 h	33 35
3	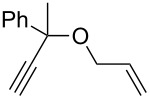	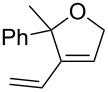	**3** **1**	1 mol %; CH_2_Cl_2_; 20 °C; 20 h	>99 >99
4	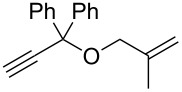	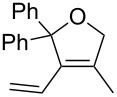	**3** **1**	5 mol %; toluene; 80 °C; 5 h	15 41
5	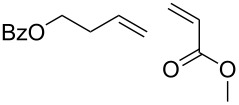 1:2	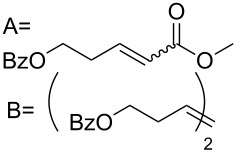	**3** **1**	1 mol %; CH_2_Cl_2_; 20 °C; 24 h	A = 30; B = 52 A = 69; B = 9
6	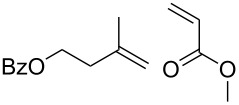 1:2	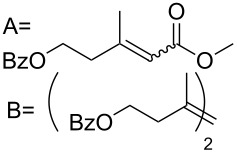	**3** **1**	5 mol %; toluene; 80 °C; 5 h	0 0
7	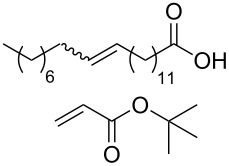 1:3	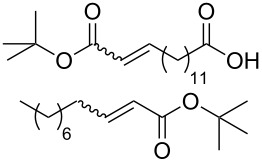	**3** **1** **3** **1**	2.5 mol %; CH_2_Cl_2_; 40 °C; 22 h 0.5 mol %; CH_2_Cl_2_; 40 °C; 17 h	>99 >99^a^ 75 81^b^
8	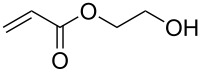	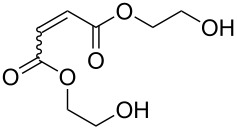	**3** **1**	2.5 mol %; CH_2_Cl_2_; 40 °C; 48 h 2.5 mol %; CH_2_Cl_2_; 40 °C; 2 h	>99 >99

^a^4% homodimer of acrylate; ^b^1% homodimer of acrylate.

Benchmark substrates were selected according to protocols for testing of metathesis catalysts [33]. Substrates with low steric hindrance (Table 3, Entry 1 and 3) were transformed with satisfying results. Even the formation of tetra-substituted olefin bonds (Table 3, Entry 2 and 4) was feasible, although yields fell short in comparison to those obtained with catalyst **1**. In cross metathesis, **3** was not particularly active in coupling terminal mono-substituted olefins with methyl acrylate and failed in the CM of di-substituted terminal olefins (Table 3, Entry 5 and 6) under the reaction conditions used. An interesting example is the cross metathesis of erucic acid with *tert*-butyl acrylate (Table 3, Entry 7). In this case, very similar results were obtained with **1** and **3**. Still a difference exists as only **1** produced small amounts of the homodimer of the acrylate. Finally, the homo-dimerisation of an acrylate was our last test reaction. Diiodo-complex **3** catalyses the dimerisation of 2-hydroxyethyl acrylate, but compared to **1,** catalyst **3** is considerably slower (Table 3, Entry 8).

## Conclusion

The results presented indicate a slight activity change in various olefin metathesis reactions when changing the anionic co-ligands from chlorides to iodides. In general, the catalysts are good for RCM and enyne metathesis of moderately hindered substrates; however, they exhibit low activity towards catalyzing transformations of sterically hindered substances. The parent dichloro derivative **1** is the most active catalyst in every transformation studied. The diiodo derivative **3** is a slightly inferior catalyst in RCM, enyne metathesis and CM, but **3** is reluctant or even ineffective to initiate ROMP of norbornene derivatives. Another example of selectivity was observed during the cross metathesis of an internal olefin with an electron deficient alkene, where in the case of **3** no side reaction (i.e., homodimerisation of the electron-deficient olefin) occurred. Thus **3** might prove in the future an interesting catalyst for special applications demanding selectivity.

The current results might be of particular importance whenever the transformation of charged substrates is of interest. In light of the easy exchange of anionic co-ligands in ruthenium-based olefin metathesis catalysts, anionic counterions should preferably be chlorides or bromides but not iodides. The latter might cause a decrease of the reaction rate or might even impede the desired transformation.

## Supporting Information

Supporting information contains full experimental and spectral data for complexes **1**–**3** and the test reactions.

File 1
