# Supplementary material for: Halide exchanged Hoveyda-type complexes in olefin metathesis
Source: Beilstein J Org Chem. 2010 Nov 23;6:1091–8. doi: 10.3762/bjoc.6.125 (PMC3002021; doi:10.3762/bjoc.6.125)
Supplement: File 1 [file Beilstein_J_Org_Chem-06-1091-s001.pdf]

Supporting Information

for

Halide exchanged Hoveyda-type complexes in olefin metathesis

Julia Wappel[1], César A. Urbina-Blanco[2], Mudassar Abbas[1], Jörg H. Albering[1], Robert Saf[1], Steven P. Nolan[2] and Christian Slugovc*[1]

[1]Institute for Chemistry and Technology of Materials (ICTM), Graz University of Technology, Stremayrgasse 9, 8010 Graz, Austria and [2]EaStCHEM School of Chemistry, University of St Andrews, St Andrews KY16 9ST, United Kingdom

Email: Julia Wappel - julia.wappel@tugraz.at; Mudassar Abbas - mabbas@student.tugraz.at; Steven P. Nolan - snolan@st-andrews.ac.uk; Christian Slugovc* - slugovc@tugraz.at

* Corresponding author

1. General

Unless otherwise noted, all reactions were carried out under an argon atmosphere in predried glassware using Schlenk techniques. Materials were obtained from commercial sources (Aldrich, Fluka or Alfa Aesar) and were used without further purification. The monomers dimethyl bicyclo[2.2.1]hept-5-ene-2,3-dicarboxylate (**4**) [1], 5,6-bis(methoxymethyl)bicyclo[2.2.1]hept-2-ene (**5**) [1], (*Z*)-cyclooct-5-ene-1,2-diyl diacetate (**6**) [2] and 1-isopropoxy-2-(prop-1-en-1-yl)benzene [3] were prepared according to literature procedures. CH_2Cl_2 was dried over CaH_2 and degassed with argon. NMR (^1H, ^{13}C) spectra were recorded on a Bruker Avance 300 MHz and an INOVA 500 MHz spectrometer, respectively in $CDCl_3$. Chemical shifts are given in

ppm relative to a $SiMe_4$ standard. The solvent peak of $CDCl_3$ was used for referencing the NMR spectra: 7.26 (^1H) and 77.16 ppm (^{13}C), respectively. Gel permeation chromatography (GPC) was used to determine molecular weights and the polydispersity index (PDI). Measurements were carried out in THF with the following arrangement: a Merck Hitachi L6000 pump, separation columns of Polymer Standards Service (5 μm grade size) and a refractive-index detector from Wyatt Technology. For calibration, polystyrene standards purchased from Polymer Standard Service were used. X-Ray measurements were performed on a Bruker AXS Kappa APEX II diffractometer using Mo Kα radiation. The structure was solved by direct methods using SHELXS and refined with SHELXL. The absorption correction was performed using the program SADABS. Field desorption mass spectra (FD-MS) were recorded on a Waters GCT Premier. Samples were dissolved in CH_2Cl_2 (c = 1 mg/mL). 0.5 µL of the solution was deposited on the FD emitter and dried under a stream of nitrogen.

2. Synthesis and characterisation of ruthenium complexes

Synthesis of (SPY-5-31)-Dichloro-(κ^2(C,O)-(2-isopropyloxybenzylidene)(1,3-bis(2,4,6-trimethylphenyl)-4,5-dihydroimidazol-2-ylidene) ruthenium (1)

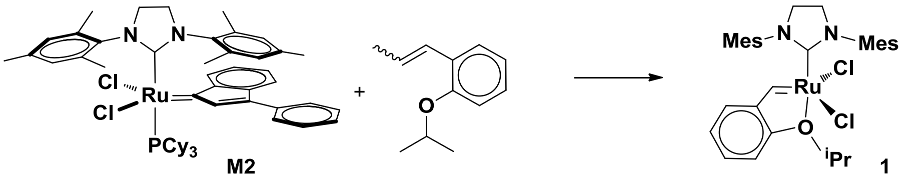

Complex **1** was prepared similarly to the published procedure [3] from **M2** and 1-isopropoxy-2-(prop-1-en-1-yl)benzene as the starting materials.

Complex **M2** (1.48 g, 1.56 mmol, 1 equiv) was dissolved in degassed, dry CH_2Cl_2 under an argon atmosphere and 1-isopropoxy-2-(prop-1-en-1-yl)benzene (0.36 g; 2.02 mmol; 1.3 equiv) and CuCl (0.19 g, 1.87 mmol, 1.2 equiv) were added. The reaction mixture was stirred at room temperature for 15 h. During this time the colour of the reaction changed from red to deep-green. The reaction progress was monitored by TLC (Cy:EtOAc 1:1). The solvent was removed in vacuo and the remaining crude product purified by column chromatography (Cy:EtOAc 15:1 then 5:1). Yield: 770 mg (78%). Analytical data were identical with those from literature [3].

^{1}H NMR (δ, 20 °C, $CDCl_3$, 300 MHz): 16.56 (s, Ru=C*H*), 7,48 (t, 1H, ph^5), 7.07 (s, 4H, C*H*$^{mes3,3'5,5'}$), 6.93 (d, 1H, ph^3), 6.85 (t,1H, ph^4), 6.80 (d,1H, ph^6), 4.92–4.87 (septet, 1H,C*H*^{isprop}), 4.18 (s, 4H, N-C*H*$_2$), 2.47 (bs, 12H, C*H*$_3^{mes7,7',9,9'}$), 2.40 (bs, 6H, s, 3H,C*H*$_3^{mes8,8'}$), 1.27 (d, 6H, C*H*$_3^{isoprop}$).

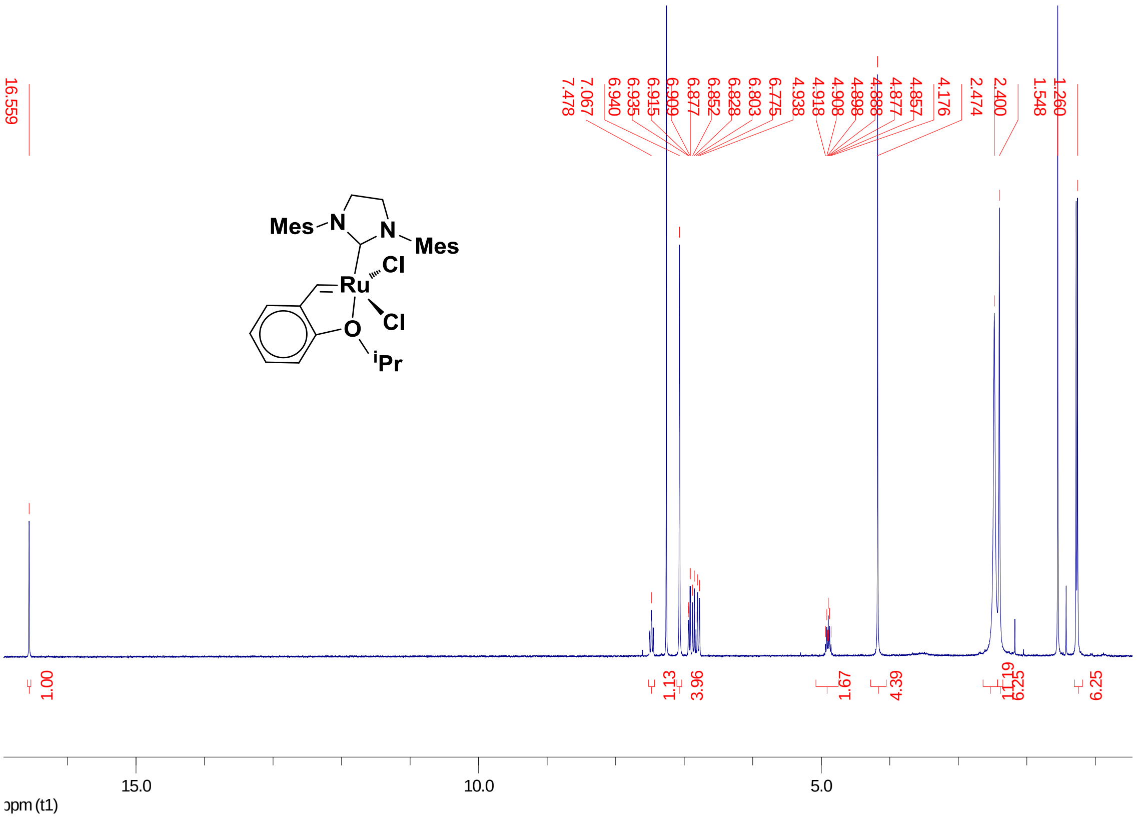

General procedure for the preparation of complexes (SPY-5-31)-dibromo-(κ^2(C,O)-(2-isopropyloxybenzylidene)(1,3-bis(2,4,6-trimethylphenyl)-4,5-dihydroimidazol-2-ylidene) ruthenium (2) and (SPY-5-31)-diiodo-(κ^2(C,O)-(2-isopropyloxybenzylidene)(1,3-bis(2,4,6-trimethylphenyl)-4,5-dihydroimidazol-2-ylidene) ruthenium (3).

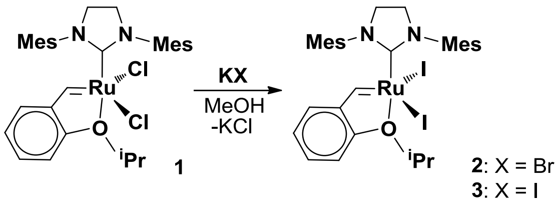

Complex **1** (300 mg, 0.471 mmol, 1 equiv) was suspended in degassed MeOH (5 mL), and KI (2.35 g; 14.13 mmol; 30 equiv) was added. The reaction mixture was stirred under an argon atmosphere for 3 h. MeOH was removed in vacuo. The

residue was extracted with degassed, dry CH_2Cl_2 (2 × 5 mL) and the dark green solution decanted into another Schlenk tube. CH_2Cl_2 was removed by evaporation and the remaining solid re-suspended in degassed MeOH (5 mL). KI (1.18 g, 7.07 mmol, 25 equiv) was added and the reaction was stirred for another 4 h. The latter procedure was repeated twice and MeOH was again removed in vacuo. The residue was extracted with CH_2Cl_2 (3 × 5 mL) and insoluble materials were removed by filtration. The green solution was evaporated to dryness and re-dissolved in CH_2Cl_2 (3 mL). Slow diffusion of diethyl ether into this CH_2Cl_2 solution, carried out under an argon atmosphere, resulted in dark green crystals, which were filtered off and dried in vacuo. Yield: 293 mg (76%).

^{1}H NMR (δ, 20 °C, $CDCl_3$, 300 MHz): 15.66 (s, 1H, Ru=C*H*), 7.53 (dd, 1H, ph^5), 7.08, 7.00 (s, 4H, C$H^{mes3,3'5,5'}$), 6.96 (d, 1H, ph^2), 6.83 (m, 2H, ph^{3,4}), 5.04 (septet, 1H,CH^{isprop}), 4.14 (s, 4H, N-CH_2), 2.68 (s, 6H, CH_3^{mes}), 2.52 (s, 6H, CH_3^{mes}), 2.44 (s, 3H, CH_3^{mes}), 2.33 (s, 3H, CH_3^{mes}), 1.45 (d, 6H, C$H_3^{isoprop}$). Characteristic signal for the mixed halide species: 16.10 (s, Ru=C*H*) (4 mol % present).

^{13}C NMR (δ, 20 °C, $CDCl_3$, 75 MHz): 298.9 (1C, Ru=*C*H), 214.2 (1*C*, Ru-*C*), 153.2, 150.1, 145.3, 139.2, 138.9, 138.7, 138.2, 137.8, 134.7, 130.6, 129.9, 129.7, 123.3, 122.0, 113.5, 75.9, 52.9, 51.2, 23.5, 21.9, 21.24, 21.17, 20.3.

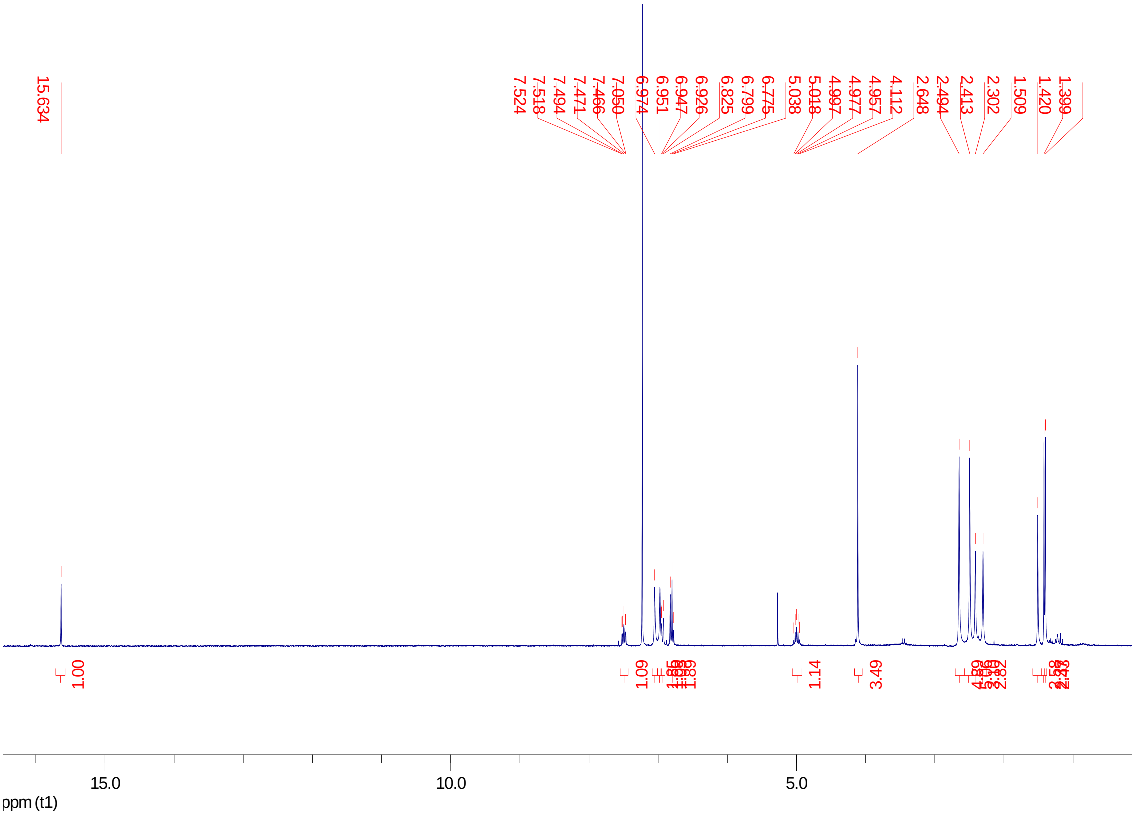

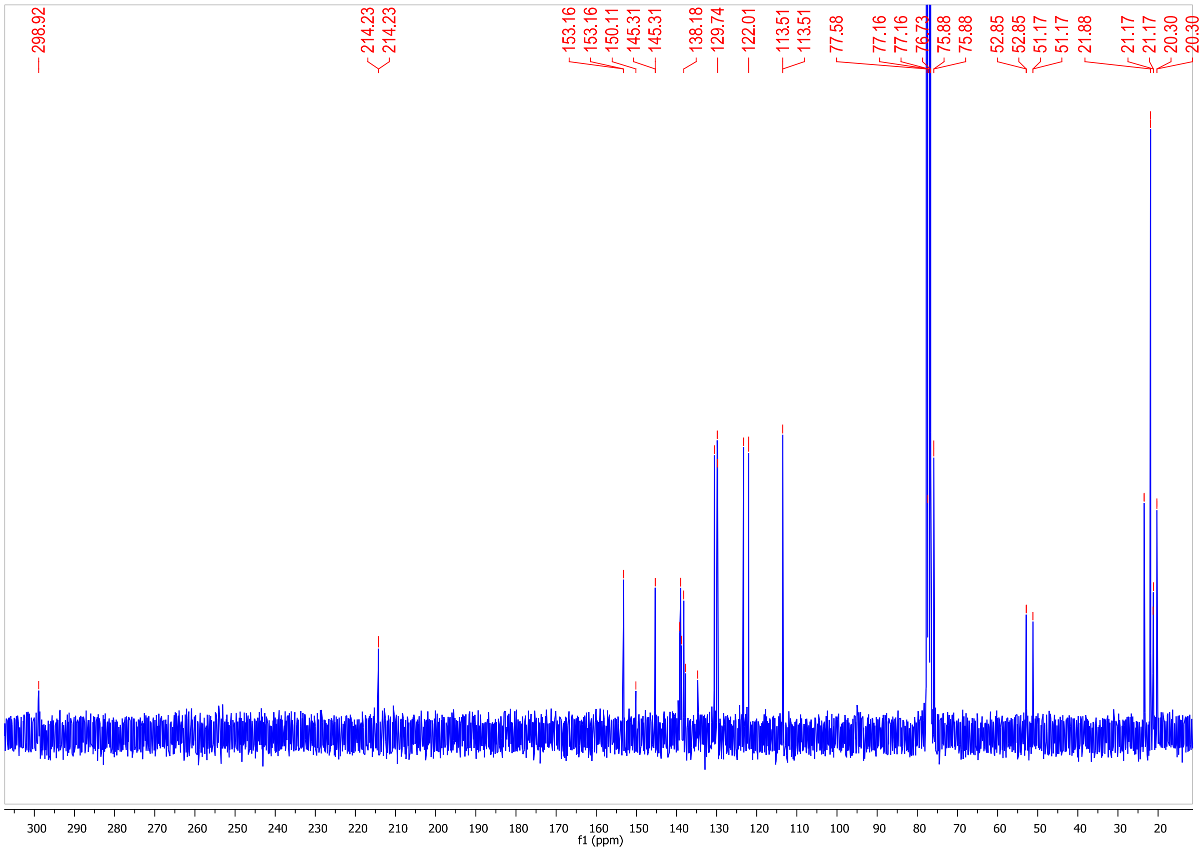

2 was prepared similarly to **3** from **1** (115 mg) and KBr (2 × 660 mg, 2 × 990 mg) instead of KI. Yield: 132 mg (91%) of a dark green microcrystalline solid.

^{1}H NMR (δ, 20 °C, $CDCl_3$, 300 MHz): 16.40 (s, Ru=C*H*), 7,52 (t, 1H, ph^5), 7.06 (s, 4H, $CH^{mes3,3'5,5'}$), 6.97 (d, 1H, ph^2), 6.88–6.80 (m, 2H, $ph^{3,4}$), 5.01–4.89 (septet, 1H, CH^{isprop}), 4.17 (s, 4H, N-CH_2), 2.60, 2.42 (bd, 18H, $CH_3^{mes7,7',8,8',9,9'}$), 1.35 (d, 6H, $CH_3^{isoprop}$). Characteristic signal for the mixed halide species: 16.48 (s, Ru=C*H*) (5 mol % present). ^{13}C NMR (δ, 20 °C, $CDCl_3$, 75 MHz): 300.1 (1C, Ru=*C*H), 212.6 (1C, Ru-*C*), 152.5 145.8, 140.0–138.1 (b), 130.1, 129.6 (b), 123.0, 122.3, 113.3, 75.5, 52.6–52.1 (b), 21.9–18.8 (b), 21.5, 21.2.

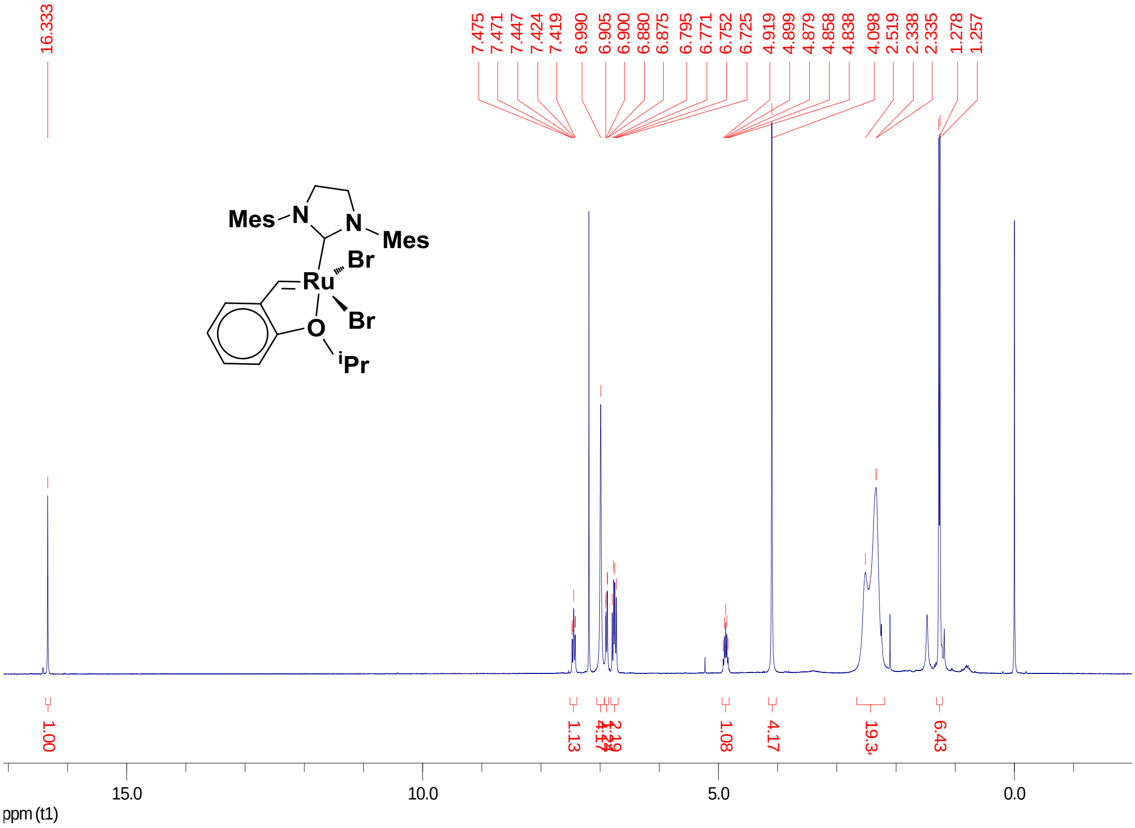
16.333
7.475
7.471
7.447
7.424
7.419
6.990
6.905
6.900
6.880
6.875
6.795
6.771
6.752
6.725
4.919
4.899
4.879
4.858
4.838
4.098
2.519
2.338
2.335
1.278
1.257
Mes
N
N
Mes
Br
Ru
Br
O
iPr
1.00
1.13
4.17
1.08
4.17
19.3
6.43
15.0
10.0
5.0
0.0
ppm (t1)

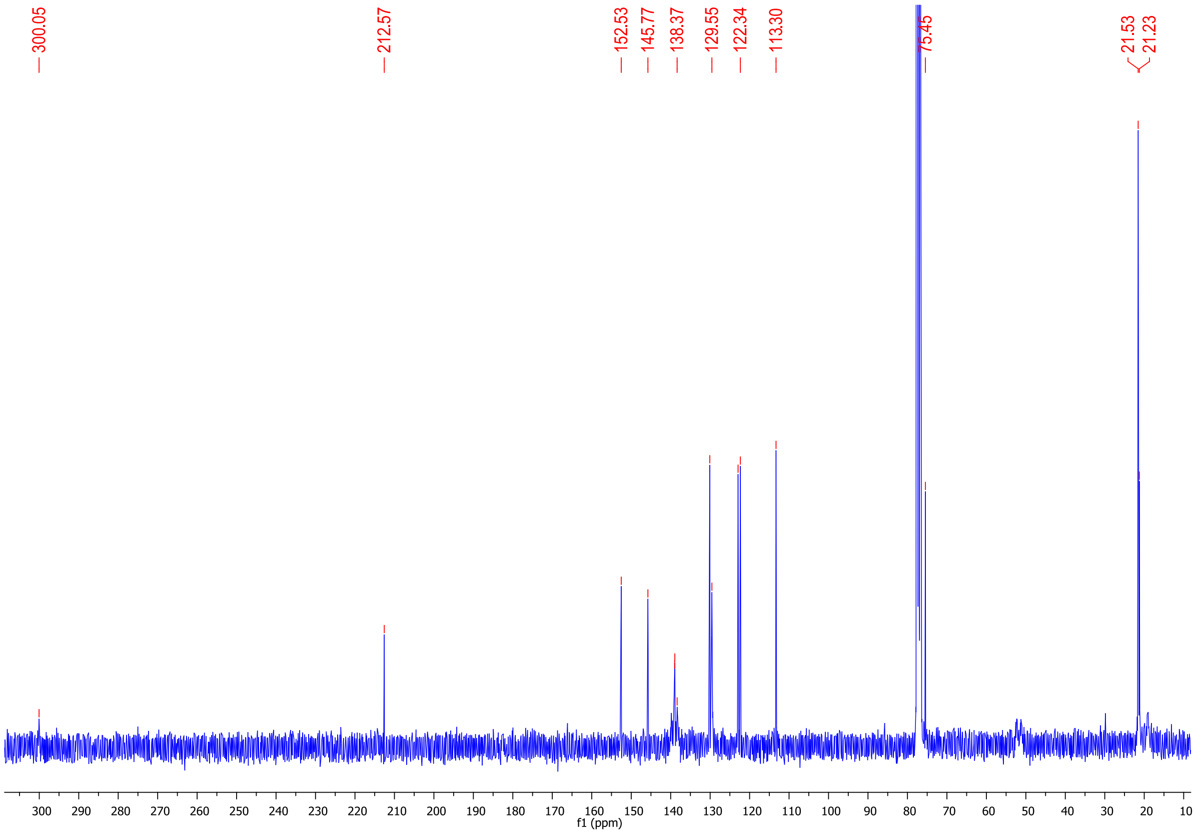
300.05
212.57
152.53
145.77
138.37
129.55
122.34
113.30
75.45
21.53
21.23
300
290
280
270
260
250
240
230
220
210
200
190
180
170
160
150
140
130
120
110
100
90
80
70
60
50
40
30
20
10
f1 (ppm)

Field desorption measurements of 2 and 3

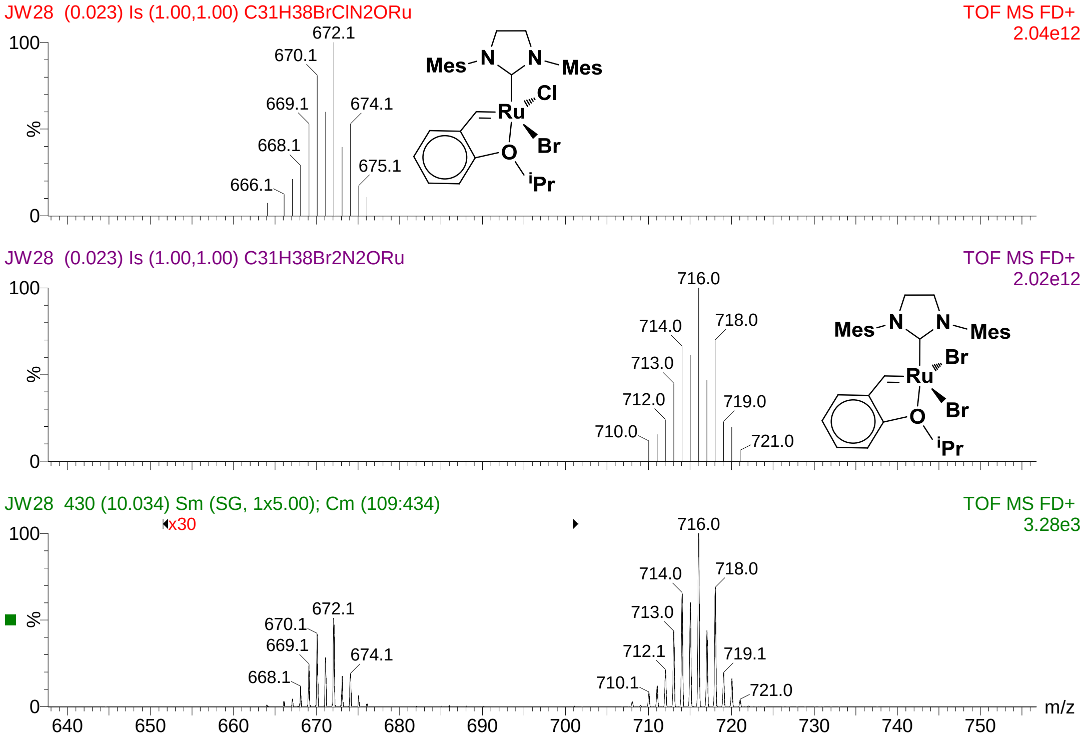

Figure S1: FD-MS spectrum of **2**; above and middle: the calculated isotope patterns; below: measured spectrum (left region 30 times magnified).

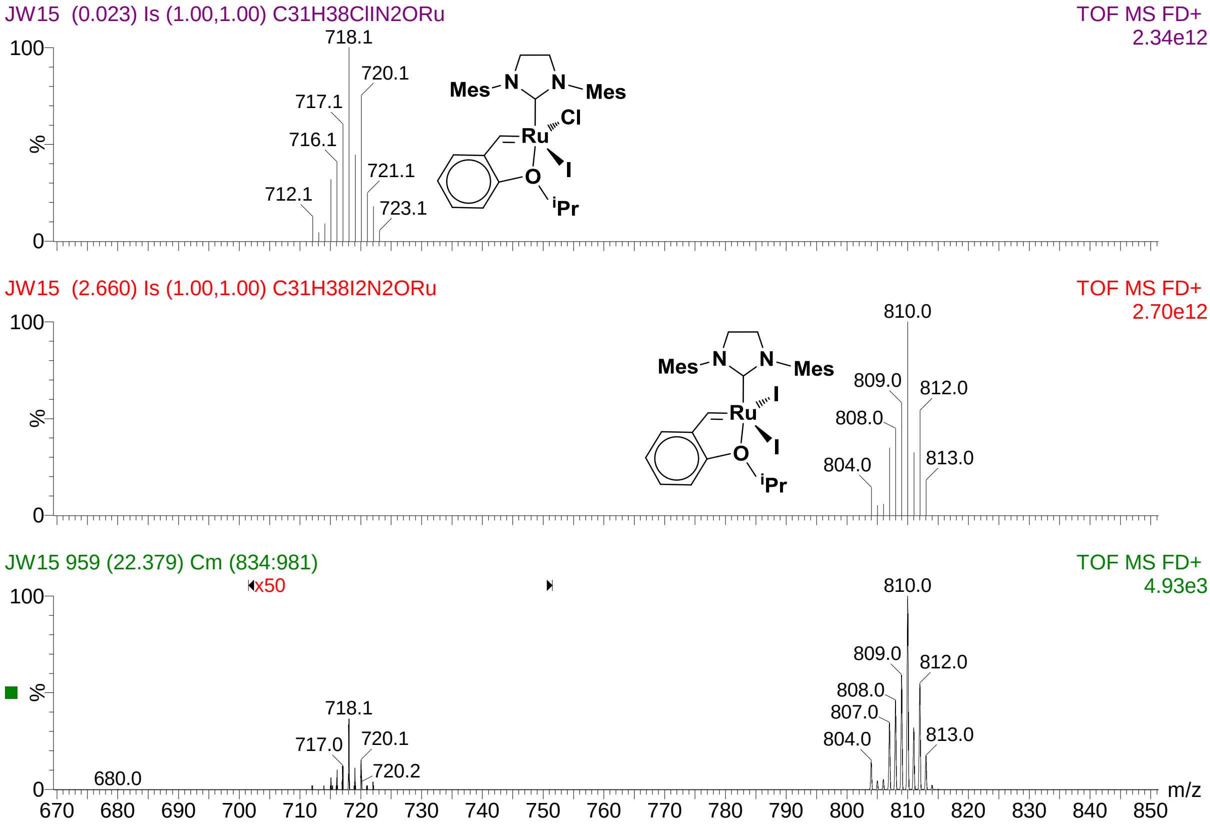

Figure S2: FD-MS spectrum of **3**; above and middle: the calculated isotope patterns; below: measured spectrum (left region 50 times magnified).

Crystal structure of 3

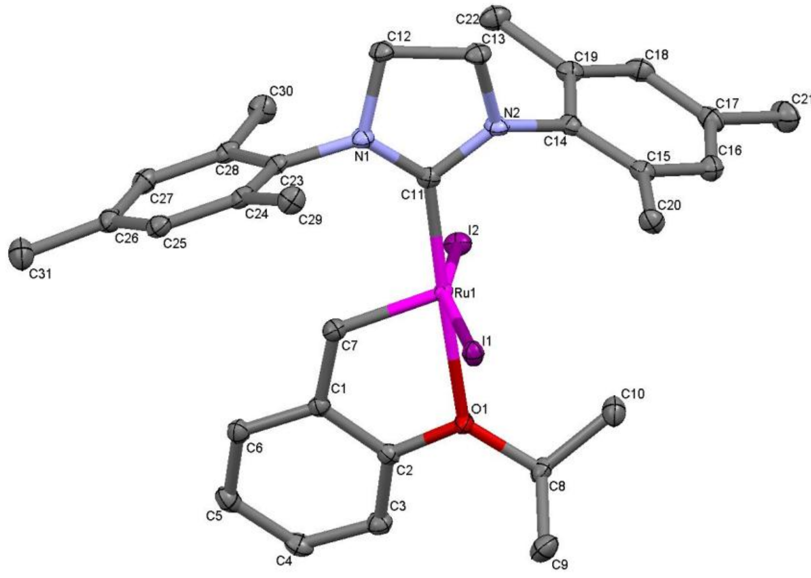

Figure S3: Crystal structure of complex **3**.

Empirical formula	C31 H38 I2 N2 O Ru	
Formula weight	809.50	
Temperature	100(2) K	
Wavelength	0.71073 Å	
Crystal system	monoclinic	
Space group	- *P* 21/*c*	
Unit cell dimensions	a = 8.9585(3) Å	α = 90°
	b = 27.5485(12) Å	β = 98.803(2)°
	c = 12.3971(6) Å	γ = 90°
Volume	3023.5(2) Å	
Z	4	
Density (calculated)	1.778 mg/m^3	
Absorption coefficient	2.587 mm^{-1}	

F(000)	1584
Crystal size	0.24 × 0.07 × 0.07 mm^3
Theta range for data collection	2.42 to 30.00°
Index ranges	−12<=h<=11, −38<=k<=37, −15<=l<=17
Reflections collected	51277
Independent reflections	8811 [R(int) = 0.0245]
Completeness to theta = 30.00°	99.9%
Absorption correction	Semi-empirical from equivalents
Max. and min. transmission	0.834 and 0.805
Refinement method	Full-matrix least-squares on F^2
Data / restraints / parameters	8811 / 0 / 343
Goodness-of-fit on F^2	1.078
Final R indices [I>2sigma(I)]	R1 = 0.0170, wR2 = 0.0390
R indices (all data)	R1 = 0.0207, wR2 = 0.0410
Largest diff. peak and hole	0.672 and −0.443 e.$Å^{-3}$

The crystal structure has been deposited at the Cambridge Crystallographic Data Centre and can be accessed using the deposition number CCDC-787338.

3. Product characterisation for RCM reactions

Diethyl cyclopent-3-ene-1,1-dicarboxlate

In an NMR tube, catalysts **1–3** (1 equiv) were dissolved in $CDCl_3$. **7** (100 equiv) dissolved in $CDCl_3$ was added. A concentration of 0.1 mol/L diethyl diallylmalonate was used. ^{1}H NMR spectra were recorded periodically and until the reaction was complete or until no further conversion of the monomers could be observed to obtain conversion time plots.

Colourless liquid. ¹H NMR (δ, 20 °C, $CDCl_3$, 300 MHz): 5.6 (s, 2H, C*H*=C*H*), 4.23–4.16 (m, 4H, CH_2-CH_3), 3.11 (s, 4H, C*H*-C), 1.24 (m, 6H, CH_3).

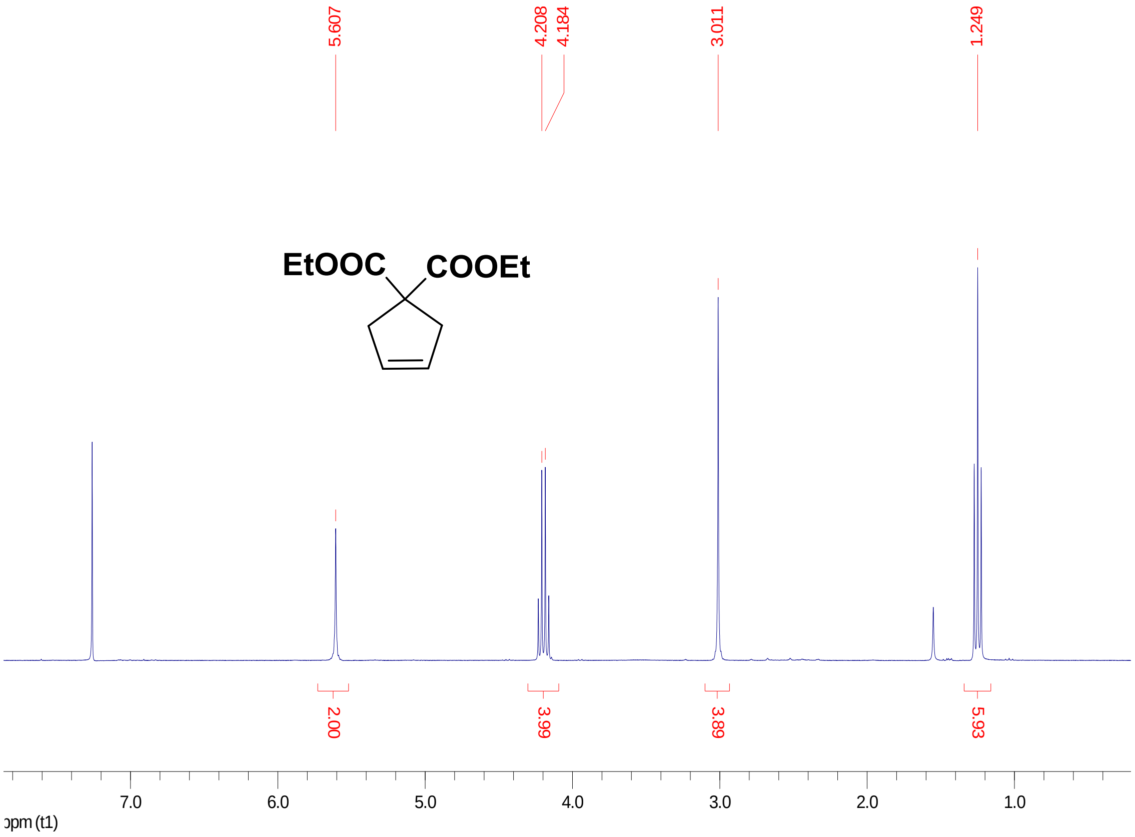

Diethyl 3,4-dimethylcyclopent-3-ene-1,1-dicarboxylate [4]

A Schlenk flask under nitrogen was charged with diethyl 2,2-bis(2 methylallyl)malonate (0.5 mmol) and toluene (5 mL, c = 0.1 M), then precatalyst **1** or **3** (5×10^{-5} mol) was added. The reaction mixture was stirred at 80 °C for 5 h. Volatiles were removed under vacuum to afford a yellow liquid. ¹H NMR yields were determined by isolation of the mixture and integration of the allylic signals.

Colourless liquid. ¹H NMR (400 MHz, $CDCl_3$): 4.20 (q, *J* = 7.12 Hz, 4H,CH_2-CH_3), 2.95 (m, 4H, CH_2-CH=), 2.90 (s, 2H, CH_2-C(CH_3)=), 1.61 (m, 6H, CH_3-C=), 1.26 (t, *J* =7.12 Hz, 6H, CH_2-CH_3).

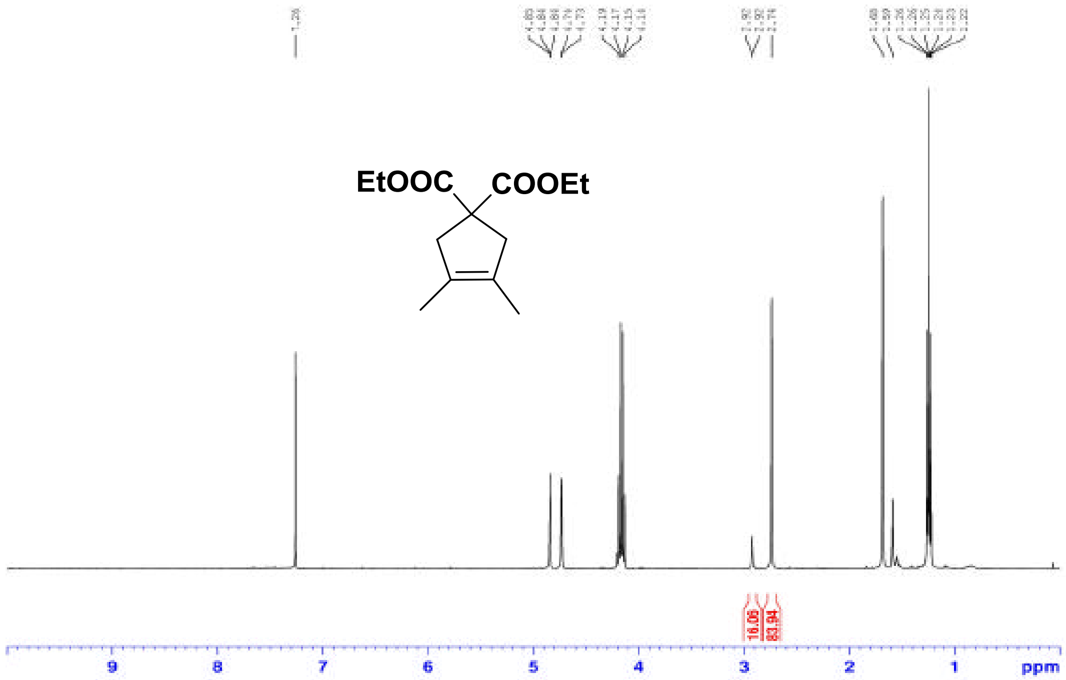

Diethyl 3-methylcyclopent-3-ene-1,1-dicarboxylate [5]

The reaction was carried out by following the procedure described above, but in CH_2Cl_2 and at room temperature.

Colourless liquid. ^{1}H NMR (400 MHz, $CDCl_3$): δ = 5.19–17 (m, 1H, C*H*=), 4.18 (q, *J* = 7.12 Hz, 4H,CH_2-CH_3), 2.97–95 (m, 2H, CH_2-CH=), 2.90 (s, 2H, CH_2-C(CH_3)=), 1.70 (m, 3H, CH_3-C=), 1.24 (t, *J* =7.12 Hz, 6H, CH2-CH_3).

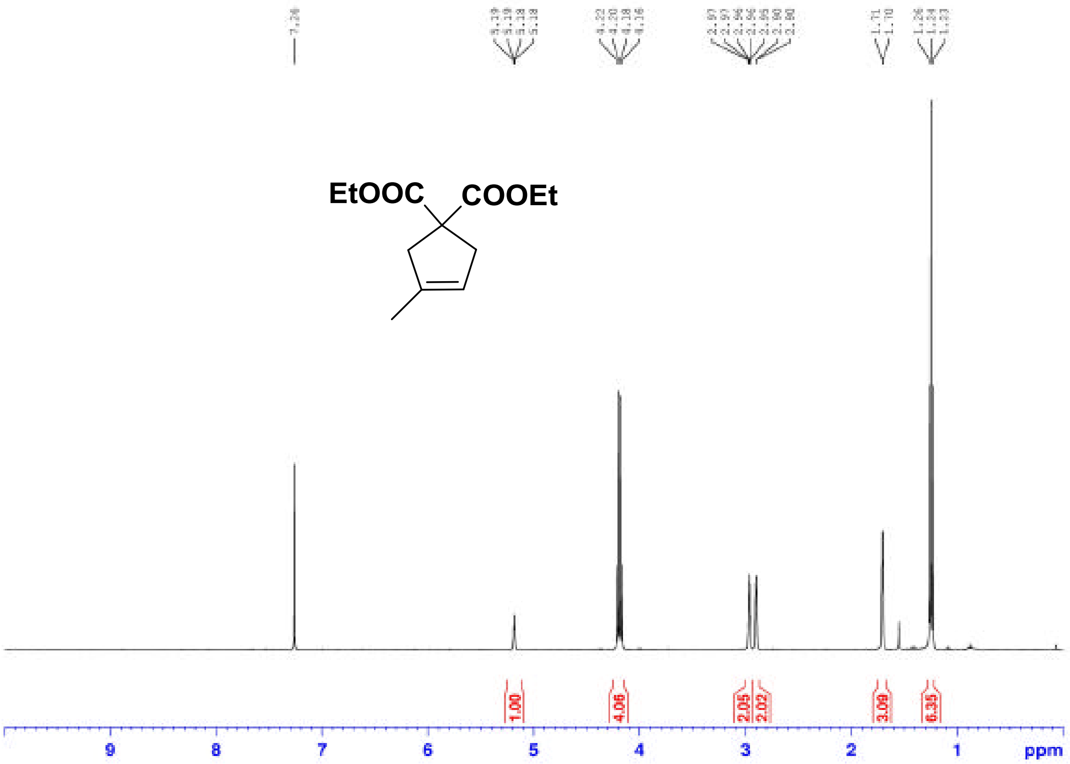

4-Methyl-2,2-diphenyl-3-vinyl-2,5-dihydro-furan [6]

A Schlenk flask under nitrogen was charged with 1,1-diphenyl-1-(2-methylallyloxy)prop-2-yne (131 mg, 0.5 mmol) and toluene (5 mL, $_c$ = 0.1 M), then precatalyst **1** or **3** (2.5 × 10–5 mol) was added. The reaction mixture was stirred at 80 °C for 5 h. Volatiles were removed under vacuum and the crude residue was purified by flash column chromatography (pentane/ether 9:1) to yield the pure product as a yellow liquid.

^{1}H NMR (400 MHz, $CDCl_3$): δ = 7.37–26 (m, 10H, H^{Ar}), 6.24 (dd, *J* = 17.7 Hz and 11.7 Hz, 1H, –CH=CH_2), 5.14 (d, *J* = 11.7 Hz, 1H, =CH_2), 5.02 (d, *J* = 17.7 Hz, 1H, =CH_2), 4.68 (s, 2H, OCH_2), 1.90 (s, 3H, CH_3).

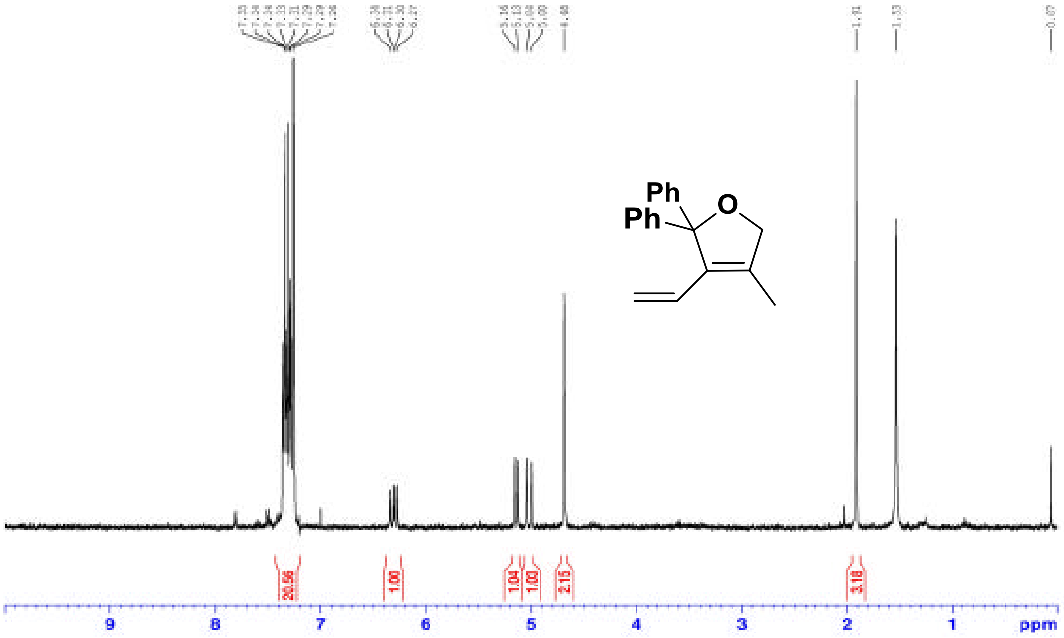

2-Methyl-2-phenyl-3-vinyl-2,5-dihydrofuran [5]

Yellow liquid. ^{1}H NMR (300 MHz, $CDCl_3$): δ = 7.44–26 (m, 5H, H^{Ar}), 6.24 (dd, *J* = 17.8 Hz and 11.2 Hz, 1H, –CH=CH2), 5.99 (s, 1H, =CH-CH_2O), 5.07 (d, *J* = 17.8 Hz, 1H, =CH_2), 5.03 (d, *J* = 11.2 Hz, 1H, =CH2) 4.77 (m, 2H, OCH_2), 1.80 (s, 3H, CH_3).

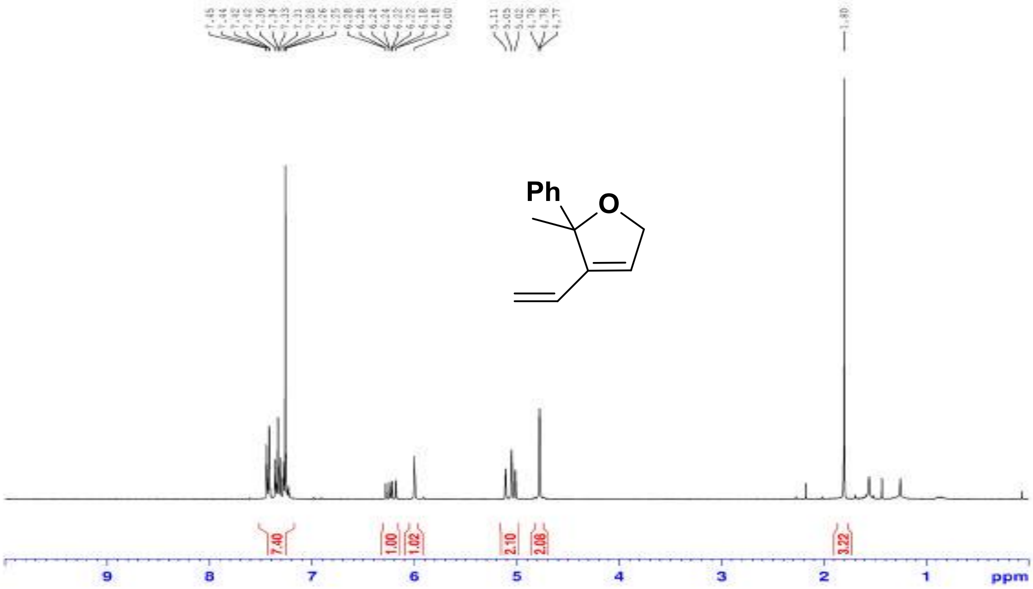

4. Product characterisation for CM reactions

A Schlenk-flask with under argon was charged with various ratios of erucic acid; *tert*-butyl acrylate and CH_2Cl_2. Precatalyst **1** and **3** (0.5 and 2.5 mol % respectively) was added. The reaction was carried out at 40 °C for several hours. Small amounts of the reaction mixture were removed from the flask from time to time, quenched with ethyl vinyl ether, concentrated in vacuo and characterised by ^1H NMR to monitor the reaction progress. Yields were not determined and the two products were not separated.

^{1}H NMR (δ, 20 °C, $CDCl_3$, 300 MHz): 6.86 (m, 2H, CH_2-C*H*=CH-COO), 5.72 (d, $^3J_{HH}$ = 15.7 Hz, 2H, CH_2-CH=C*H*-COO), 2.33 (t, 2H, C*H*$_2$-C=O^B), 2.16 (m, 4H, C*H*$_2$-CH^{A+B}), 1.62 (m, 2H, C*H*$_2$-CH_2-C=O^B), 1.48 (s, 18H, C*H*$_3$-C^{A+B}), 1.26 (bs, 24H, C*H*$_2$$^{A+B}$), 0.87 (t, 3H, C*H*$_3$-$CH_2$^{A}). 6 % *cis* isomers as indicated by the peak: 6.09 (m, 1H, CH_2-C*H*=CH-COO).

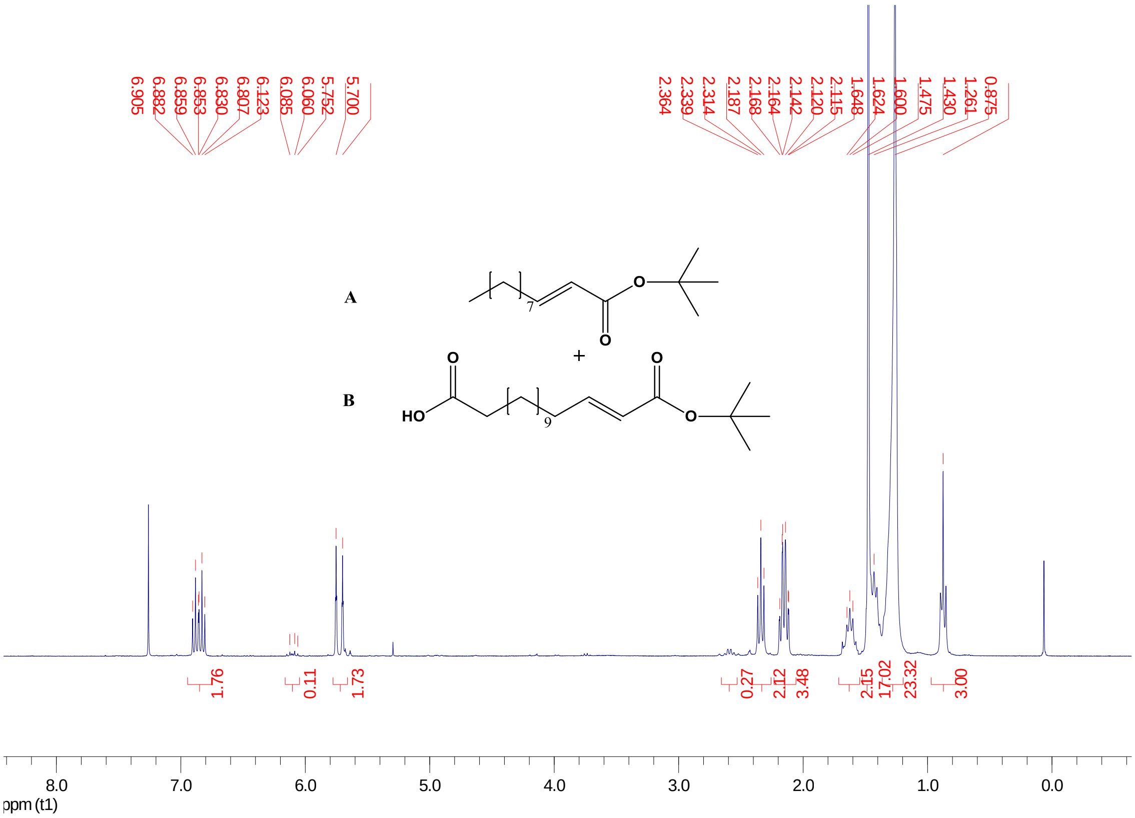

(*E*)-5-Methoxy-5-oxopent-3-enyl benzoate

White solid. ^{1}H NMR (400 MHz, CDCl3): δ = 8.04–8.01 (m, 2H, HAr), 7.58–7.54 (m, ^{1}H, HAr), 7.46–7.42 (m, 2H, HAr), 7.01 (dt, *J* = 15.7 and 7.0 Hz, 1H, HC=), 5.97 (dt, *J* = 15.7 and 1.5 Hz, 1H, =CH-CO), 4.43 (t, *J* = 6.5 Hz, 2H, CH2-O), 3.73 (s, 3H, CH3), 2.71–2.66 (m, 2H, C*H*2-CH=).

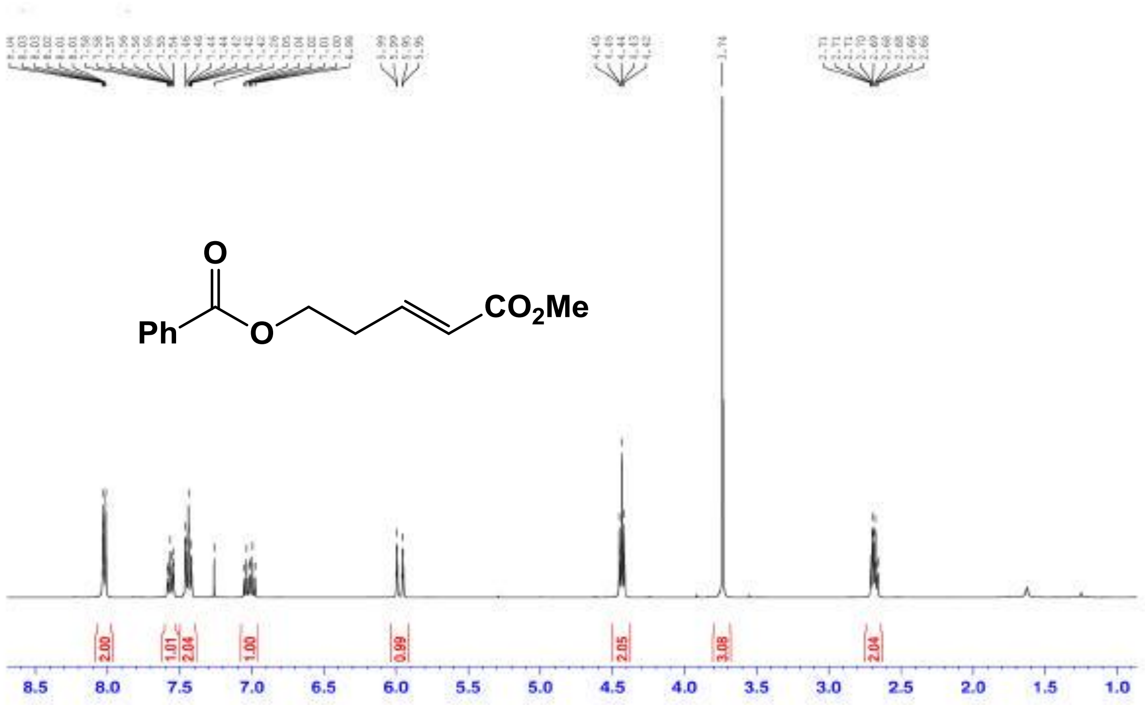

5. **Product characterisation for ROMP products**

General procedure for polymer kinetic measurements

The same procedure was used as that for the kinetic measurements of the RCM. The ratio of initiator to **4** was 1:50.

General polymerisation procedure for polymers

Solutions of complex **1–3** (1 equiv) and **4, 5** and **6**, respectively (300 equiv, 0.02 mmol/mL) and **6** were prepared. Polymerisation was carried out in CH_2Cl_2 at room temperature under an inert atmosphere of argon. After complete consumption of the monomer, the reaction was stopped by the addition of an excess of ethyl vinyl ether (50 equiv). The polymer was precipitated in vigorously stirred methanol

(approx. 15 mL for 100 mg polymer), and the white to yellowish precipitate was sampled and dried in vacuum. Yield: 97–20%.

poly4: ^{1}H NMR (δ, 20 °C, $CDCl_3$, 300 MHz): 5.50–5.19 (bm, 2H, C*H*=C*H*), 3.63 (bd, 6H, $CH_3^{5,5'}$), 3.24–2.97 (bd, 4H, $CH^{2,2',3,3'}$), 1.97, 1.47 (bs, 2H,CH^{1}).

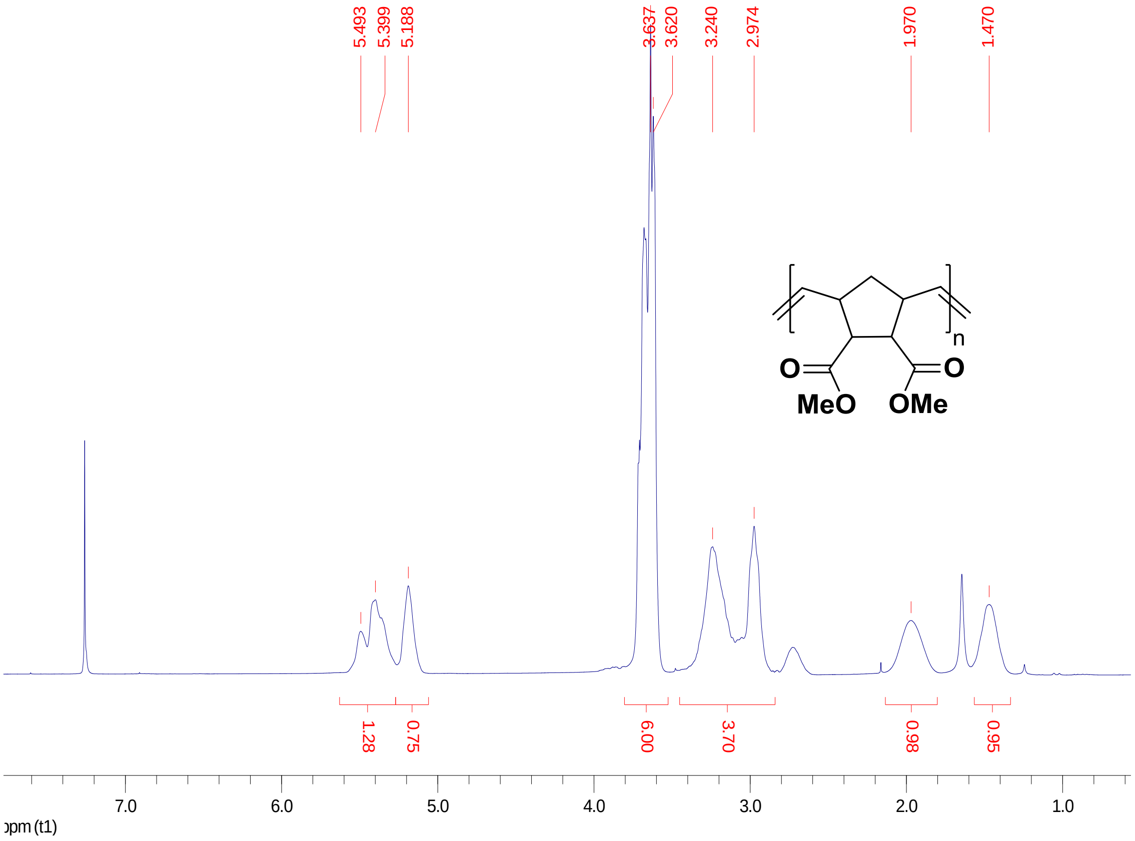

poly5: ^{1}**H NMR** (δ, 20 °C, $CDCl_3$, 300 MHz): 5.34 (bm, 2H, $CH{=}CH$), 3.31 (d, 10H, $CH_2^{4,4}$ bzw. $CH_3^{5,5'}$), 3.0, 2.64, 2.10, 1.80 (bs), 1.35 (bm, 6H, $CH^{2,2',3,3'}$ bzw. CH_2^{1}).

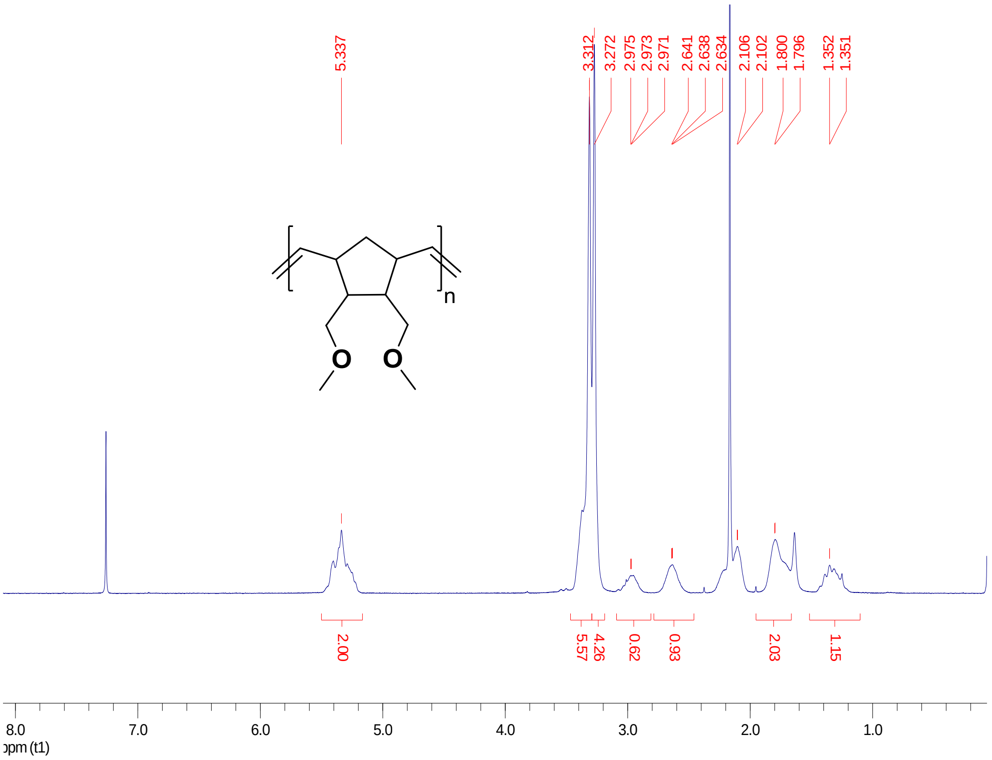

poly6: ^{1}**H NMR** (δ, 20 °C, $CDCl_3$, 300 MHz):5.34–5.30 (bm, 2H, $CH{=}$), 5.00–4.97 (bm, 2H, CH-CH_2), 2.08 (d, 6H, CH_3), 2.02–1.95, 1.62–1.53 (bm, 8H, CH_2-CH.

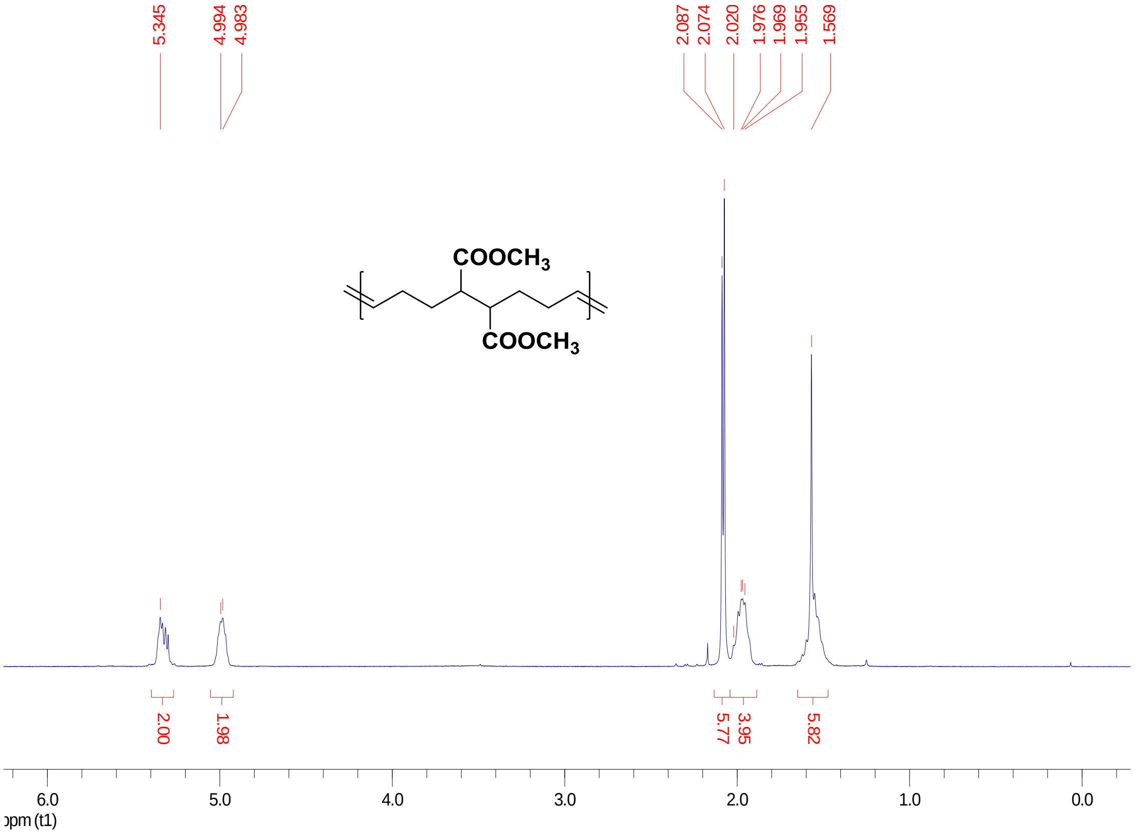

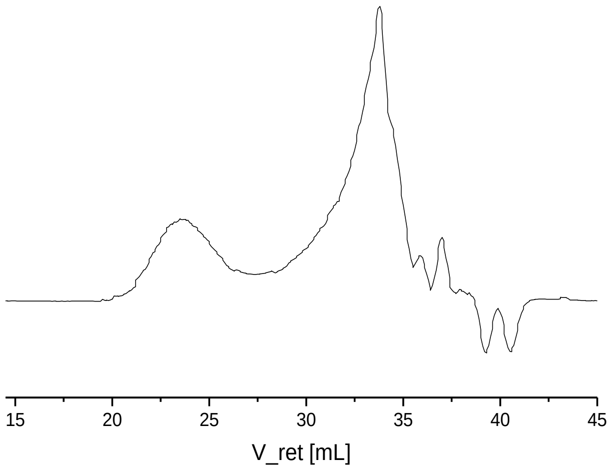

Figure S4: GPC measurement of **poly6** initiated by **3** – after 22 h reaction time – left peak: remaining polymer with high molecular weight – right peak decomposed polymer (oligomers).

References

1. Devine, P. N.; Oh T. *J. Org. Chem*. **1992,** *57,* 397. doi:10.1021/jo00027a074
2. Wang, Z.; Cui, Y. T.; Xu, Z. B.; Qu, J. *J. Org. Chem.* **2008,** *73,* 2270–2274. doi:10.1021/jo702401t
3. Garber, S. B.; Kingsbury, J. S.; Gray, B. L.; Hoveyda, A. H. *J. Am. Chem. Soc.* **2000,** *122,* 8168–8179. doi:10.1021/ja001179g
4. Ritter, T.; Hejl, A.; Wenzel, A. G.; Funk, T. W.; Grubbs, R. H. *Organometallics* **2006,** *25,* 5740–5745. doi:10.1021/om060520o
5. Clavier, H.; Nolan, S. P. *Chem. Eur. J.* **2007,** *13,* 8029–8036. doi:10.1002/chem.200700256
6. Fürstner, A.; Ackermann, L.; Gabor, B.; Goddard, R.; Lehmann, C. W.; Mynott, R.; Stelzer, F.; Thiel, O. R. *Chem. Eur. J.* **2001,** *7,* 3236–3253. doi:10.1002/1521-3765(20010803)7:15<3236::AID-CHEM3236>3.0.CO;2-S
